# Clinical utility of indigenously formulated single-vial lyophilized HYNIC-TOC kit in evaluating Gastro-entero Pancreatic neuro endocrine tumours

**Published:** 2014

**Authors:** Ajit S Shinto, K Kamaleshwaran, K Vyshak, Natarajan Sudhakar, Sharmila Banerjee, Aruna Korde, Grace Samuel, Madhav Mallia

**Affiliations:** 1 Nuclear Medicine Department, Kovai Medical Center and Hospital, India; 2 Isotope Applications & Radiopharmaceuticals Division, Bhabha Atomic Research Centre, Mumbai, India

**Keywords:** HYNIC TOC, Neuroendocrine tumor, SPECT CT

## Abstract

**Objective(s):**

The objective of this study was to evaluate the performance and utility of ^99m^Tc HYNIC-TOC planar scintigraphy and SPECT/CT in the diagnosis, staging and management of gastroenteropancreatic neuroendocrine tumors (GPNETs).

**Methods:**

22 patients (median age, 46 years) with histologically proven gastro- entero- pancreatic NETs underwent ^99m^Tc HYNIC-TOC whole body scintigraphy and regional SPECT/CT as indicated. Scanning was performed after injection of 370-550 MBq (10-15 mCi) of ^99m^Tc HYNIC-TOC intravenously. Images were evaluated by two experienced nuclear medicine physicians both qualitatively as well as semi quantitatively (tumor to background and tumor to normal liver ratios on SPECT -CT images). Results of SPECT/CT were compared with the results of conventional imaging. Histopathology results and follow-up somatostatin receptor scintigraphy with ^99m^Tc HYNIC TOC or conventional imaging with biochemical markers were considered to be the reference standards.

**Results:**

^99m^Tc HYNIC TOC showed sensitivity and specificity of 87.5% and 85.7%, respectively, for primary tumor and 100% and 86% for metastases. It was better than conventional imaging modalities for the detection of both primary tumor (*P*<0.001) and metastases (*P*<0.0001). It changed the management strategy in 6 patients (31.8%) and supported management decisions in 8 patients (36.3%).

**Conclusion:**

^99m^Tc HYNIC TOC SPECT/CT appears to be a highly sensitive and specific modality for the detection and staging of GPNETs. It is better than conventional imaging for the evaluation of GPNETs and can have a significant impact on patient management and planning further therapeutic options.

## Introduction

Neuroendocrine tumors (NETs), generally considered to be an uncommon entity of neoplasms originating from the neural crest, located mainly in the gastroenteropancreatic tract and lung; with occasional reports of ovarian and thymic origin (1). GPNETs though generally slow-growing, with an asymptomatic and indolent course over many years, many GPNETs are malignant and the patients can present with symptoms related to the primary tumor mass or metastases (2-4). Metastatic tumors have a poorer prognosis with a limited survival (5-7). Complete surgical excision is the treatment of choice for patients with NET, but in many patients such treatment is not possible at diagnosis; with liver being the most common site of metastases (8-10). On a case by case basis, various palliative treatments are offered, such as surgical debulking where the symptoms are related to tumors, chemotherapy or interferons for systemic control, intra arterial chemo or radio embolisation for hepatic metastases. However these treatments have only a limited role in symptomatic improvement and in partial tumor regression (11, 12).

This is an area where somatostatin analogs have played a significant role in not only diagnosis and staging, but also in identifying a potential molecular level target which can be utilized for therapeutic purposes. Thus, high-level of somatostatin receptor expression makes NETs amenable to treatment using peptide receptor radionuclide therapy in surgically inoperable cases and in advanced cases (13). Conventional imaging modalities such as CT, ultrasound and MRI have largely been replaced by Somatostatin receptor scintigraphy (SSTRI) for both staging, therapy planning and even for follow up imaging (14). Somatostatin, a cyclic peptide hormone consisting of 14 amino acids, acts on tissues through specific receptors that are present in the central nervous system, gastrointestinal tract, and on many cells of neuroendocrine and non-neuroendocrine origin (15).

Overexpression of these receptors has been identified in a wide range of malignant neoplasms. The expression of somatostatin receptors by NETs has led to the use of peptide receptor scintigraphy with radiolabeled somatostatin analogs for imaging. Somatostatin analogues are original hormone molecules which have been modified in various ways, usually in a form shortened to eight amino acids (octapeptides), while preserving most of the biological activity of the original hormone and are less susceptible to enzymatic degradation. They are applicable for diagnostic purposes, as they are stable much longer *in vivo* and are metabolically stable in the human body (their half-life may reach several hours) (16). At present, five types of somatostatin receptors (SSTR) have been identified. The native somatostatin shows affinity for all of them; the analogues, however, show substantial variation in this respect (17).

In recent years, attempts have been made to develop an analogue with an affinity for all receptor types because, at least one subtype is over expressed in the majority of tumors (18). There is an extensive amount of data in the literature demonstrating that the scintigraphic imaging of somatostatin positive tumors is possible using Octreoscan (Mallinckrodt, Petten, The Netherlands), that is, a conjugate of octreotide labelled with ^111^In -DTPA. This tracer enables the scintigraphic imaging of neuroendocrine tumors, which are difficult to detect with other imaging modalities (19, 20). However, the use of this radiopharmaceutical has substantial drawbacks: (1) the high cost of the cyclotron produced radionuclide, as well as its suboptimal physical characteristics and (2) the energy of emitted gamma rays makes it suboptimal for scintigraphy. In addition, the physical half-life of 68 hours of ^111^In causes a relatively high radiation exposure of patients and has the drawback of limited resolution of SPECT technology and uneven biodistribution of the radiotracer in the liver and spleen, obscuring small lesions.

Attempts to eliminate these drawbacks led, a few years ago, to the synthesis of depreotide, another small peptide, which, in contrast to octapeptides, can be directly labeled with ^99m^Tc. This radiopharmaceutical, known under the commercial name of Neotect (Schering), is now being used mostly for differential diagnostics of malignant from benign solitary pulmonary nodules (21-23). Availability of ^68^ Ga isotope, a generator-derived positron-emitting radionuclide that can be easily labeled to macrocyclic chelators such as DOTA (1, 4, 7, 10-tetraaz-acyclododecane-1, 4, 7, 10-tetraacetic acid) has made possible the development of new PET radiotracers for the detection of somatostatin receptors with greater specificity.

Recently, numerous communications on preclinical studies and clinical trials of other ^99m^Tc labeled somatostatin analogues appeared; mostly octapeptides bound to ^99m^Tc via special chelators (24-33). All of these studies were aimed at a radiopharmaceutical showing a high affinity for the maximum number of receptor subtypes, as well as optimal pharmacokinetic characteristics. A new somatostatin analogue applied in this study, a Tyr3-octreotide (TOC) labeled with ^99m^Tc has been developed by Maecke and Béhé for its favorable preclinical characteristics. These preclinical data were confirmed by Decristoforo and Mather, and their early clinical studies recently appeared (33, 34). We have utilized the newly developed lyophilised kits from Bhabha Atomic Research Centre (BARC) for the preparation of ^99m^Tc Hynic TOC and report on their utility in GPNETS.

The aim of this study was to assess the performance and obtain indications on the potential clinical usefulness of scintigraphy with the new ^99m^Tc Hynic TOC radiopharmaceutical in GPNET’s. The more specific purpose was to see whether it is possible to detect primary tumors and metastases of somatostatin receptor positive tumors and utilize it for therapy planning with ^177^Lu labeled peptides.

## Methods

### Subjects

This was a prospective study. A total of 22 patients with histologically proven GPNETs were evaluated with ^99m^Tc Hynic TOCSPECT/CT between October 2012 and April 2013. Only the staging planar and SPECT/CT studies were used for the analysis; follow-up studies were excluded. This study was conducted in accordance with our institute’s ethics protocol after obtaining ethics committee approval and written informed consent was obtained from all patients. The results of ^99m^Tc Hynic TOC planar and SPECT/CT were analyzed from the following perspectives: staging disease in patients with already diagnosed NET, detecting sites of recurrence in patients with treated NET (restaging), diagnosing NET in patients suspected of having NET on the basis of clinical features or biochemical results, comparing SPECT/CT results with the results of conventional imaging modalities, selecting potential candidates for peptide-based radioreceptor therapy (PRRT), and establishing whether the results of ^99m^Tc Hynic TOC SPECT/CT have an impact from a management point of view.

### Radiopharmaceutical Preparation

Lyophilized Hynic TOC kit vials were obtained as gift from Radiopharmaceuticals Division, Bhabha Atomic Research Centre (Mumbai). Detailed procedure for the preparation of Hynic TOC kit will be reported elsewhere. Each lyophilized kit vial contains Hynic TOC (30 µg), EDDA (10 mg), tricine (20 mg), sodium phosphate dibasic heptahydrate (4.5 mg), sodium phosphate monobasic (1 mg) and stannous chloride dehydrate (40 µg). The lyophilized kits were subjected to thorough quality control checks, including bacterial endotoxin test and sterility test, at the manufacturer’s end before releasing for clinical use.

The radiolabeling procedure was performed under aseptic conditions. ^99m^Tc-Hynic TOC was prepared by adding 10 - 60 mCi (370 - 2220 MBq) of freshly eluted sterile ^99m^Tc-sodium pertechnetate in 1-3 mL of saline, obtained from ^99^Mo/^99m^Tc alumina column generator (Radioisotope Centre POLATOM), into the kit vial and heating the vial in boiling water bath for 20 min.

Quality control of the preparation was performed using ITLC-SG (Varian USA) About 2 µL of the test solution was spotted on two separate ITLC-SG strips (10 cm long) at 1.5 cm from the bottom of the strip. One of the strips was developed in methyl ethyl ketone (Strip A) in which pertechnetate moves with the solvent front while ^99m^Tc-Hynic TOC and reduced Technetium (^99m^TcO_2_) remains at the point of spotting. The other strip was developed in acetonitrile: water (1:1, v/v) (Strip B) where the ^99m^Tc-Hynic TOC as well as pertechnetate moves with the solvent front while reduced Technetium remains at the point of spotting. When the developing solvent front reaches about 1 cm from the top of the strip, the strips were removed from the TLC jar, dried, cut into two equal segments and activity associated with each segment was determined in a NaI(Tl) counter with the energy window set for ^99m^Tc. %RCP was determined using the following equation where;

A_top_=Activity of strip A top segment,

A_total_=Total activity of strip A,

B_bot_=Activity of strip B bottom segment,

B_total_=Total activity of strip B.

% RCP=100–[(A_top_/A_total_)+(B_bot_/B_total_)]

The labeling yield in all cases exceeded 90%. The radiochemical purity determined in any of the procedures varied between 92% and 98%, with the free pertechnetate content in the range of 1.54% to 2.93%. Sterility and pyrogenicity testing was done by the supplier (BARC) prior to despatch of the kits to our centre.

### Imaging Protocols

Somatostatin analogues were stopped in patients receiving cold analog therapy before imaging: short-acting analogs for 3 days before scanning and long-acting analogs for 4–6 weeks. Fasting was not mandatory.

Each patient was administered the radiopharmaceutical of activity between 740 MBq and 925 MBq, which corresponded to 20 micrograms of Hynic TOC. The imaging was started 1.5 hours post injection and was repeated in selected cases after another 2 hours. Repetition was prompted in case there were difficulties in the evaluation of the abdominal region, which usually resulted from the elimination of a part of the complex via the gastrointestinal (GI) tract. A whole-body scan and a single photon emission computed tomography (SPECT) acquisition in the region where the tumor was located (e.g., head, thorax, abdomen) was acquired. A dose of 370-550 MBq (10–15 mCi) of ^99m^Tc-Hynic TOC was injected IV. After a 60 to 90 minutes uptake period, patients underwent whole body scan followed by a regional SPECT/CT of the primary and metastatic sites. No contrast agent was used. The whole body images were acquired at a table speed of 8 centimeters per minute in a dual headed gamma camera (Siemens Symbia T equipped with an LEHR collimator, with energy peak of 140 Kev±15% window level).

In the SPECT/CT system, the CT acquisition was performed with a slice thickness of 4 mm and a pitch of 1 on a helical dual-slice CT unit. Images were acquired using a matrix of 512×512 pixels and a pixel size of 1 mm. Additional spot views were obtained when necessary. SPECT data were acquired using a matrix of 128×128 pixels and a slice thickness of 1.5 mm. CT-based attenuation correction of the emission images was used. SPECT images were reconstructed by iterative method ordered subset expectation maximization (2 iterations and 8 subsets). After completion of SPECT acquisition, the reconstructed attenuation corrected SPECT images, CT images, and fused images of matching pairs of SPECT and CT images were available for review in the axial, coronal, and sagital planes, as well as in maximum intensity projections and in the 3D cine mode.

### Image Analysis

^99m^Tc-Hynic TOC planar and SPECT/CT studies were evaluated by two experienced nuclear medicine physicians in consensus. They were blinded to the findings of structural imaging. They evaluated the ^99m^Tc-Hynic TOC images both qualitatively and semi quantitatively. Any non physiologic focal area of increased ^99m^Tc-Hynic TOC uptake was sought. For ^99m^Tc-Hynic TOC, any non physiologic uptake that was greater than uptake in the surrounding area was interpreted as a positive finding for NET. Positive findings on ^99m^Tc-Hynic TOC were localized to anatomic images from the unenhanced CT study. The criteria for a correct detection of NET by ^99m^Tc-Hynic TOC are both positive ^99m^Tc-Hynic TOC uptakes and the correct anatomic localization of the tumor. The SPECT/CT findings were grouped as primary or metastatic disease. The maximum Tumor to background and tumor to normal liver ratios of primary lesions and metastatic lesions were calculated. For final analysis, the lesion with the highest pathologic tracer accumulation within each region in each patient was recorded.

### Reference Standard

Histopathology results and the results of conventional imaging and follow-up imaging in combination with biochemical markers were used as the reference standard. Histopathologic confirmation was done for the primary site in most of the patients, which was not possible in all metastatic lesions because of technical or ethical reasons. Hence, few of the primary lesions and most of the metastatic lesions detected on ^99m^Tc-Hynic TOC were confirmed with conventional imaging and follow-up data, with ultrasound, or in the case of pancreatic lesions, with endoscopic ultrasound and biochemical markers. Conventional imaging modalities (contrast enhanced CT, MRI and ultrasound) reports were retrieved from the hospital database and reviewed for comparison.

### Statistical Analysis

Continuous variables were expressed as medians and ranges. Categoric data were expressed as numbers and percentages. The sensitivity, specificity, accuracy, positive predictive value (PPV), and negative predictive value (NPV) of ^99m^Tc-Hynic TOC and conventional imaging modalities for detecting primary and metastatic lesions were calculated. The McNemar test was used to compare the diagnostic accuracy of ^99m^Tc-Hynic TOC with conventional imaging modalities. The Mann-Whitney test (two-tailed) was used to compare continuous variables. A *P* value of less than 0.05 was considered to be statistically significant. All the data analyses were performed using a statistical software package (SPSS, version 11.5, SPSS).

## Results

### Patient Characteristics

A total of 22 patients with known gastro-entero-pancreatic NET were included in the current study. Patient characteristics including age, sex, serum chromogranin a level, histology result, treatment received, and indication for scintigraphy are detailed in Table 1. The most common histopathologic variant was carcinoid tumor followed by pancreatic NET.

**Table 1:** Patient and disease characteristics

	Variable	Value	Patients %
**Age (y)**	Median	48	Not applicable
	Range	36 to 67	
**Sex (no. of patients)**	Male	14	63.6
	Female	8	36.4
**Chromogranin A (ng/mL)**	Median	468	Not applicable
	Range	118-5400	
**Diagnosis**	Carcinoid	11	50
	Pancreatic NET	6	27.3
	Gastrinoma	1	4.55
	Neuroendocrine tumor NOS	4	18.1
**Treatment**	Surgery	2	9.1
	Octreotide	6	27.3
	Surgery and octreotide	4	18.1
	Surgery and chemotherapy, radiotherapy, or both	2	9.1
	Octreotide and chemotherapy	3	13.6
	None	5	22.7
**Indication for scintigraphy**	Staging	8	36.3
	Restaging	14	63.7

### Reference Standard

The reference standard for considering a lesion detected on ^99m^Tc-Hynic TOC planar or SPECT/CT as either positive or negative for NET was based on a combination of the followings: histopathology results of the primary tumor in 16 patients (biopsy, n=10; fine-needle aspiration, n=6 patients), histopathology of metastatic lesions in 14 patients (biopsy, n=4 fine-needle aspiration, n=10 patients), follow-up ^99m^Tc-Hynic TOC planar or SPECT/CT in 6 patients, correlation with conventional imaging (contrast enhanced CT or MRI) in 21 patients, biochemical marker in 19 patients, endoscopic ultrasound in 7 patients, and upper gastrointestinal endoscopy in 5 patients. Hence, in some patients, more than one method was used to confirm the diagnosis. Based on the reference standard, 16 patients had primary tumor and all 22 patients had metastases.

### ^99m^Tc-Hynic TOC planar or SPECT/CT for Primary Tumor

**Figure 1:**
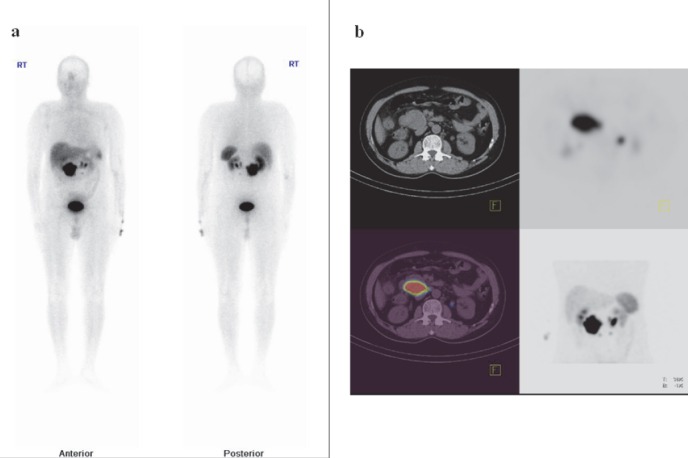
a) Whole body anterior and posterior Tc-99m-HYNIC-TOC images showing primary pancreatic neuroendocrine tumour. b) SPECT, CT and fused SPECT/CT slices localized uptake to the pancreatic tumour

^99m^Tc-Hynic TOC planar or SPECT/CT detected primary tumor in 14 of 16 patients who had primary tumor according to the reference standard. A total of 15 primary tumors were localized in these 14 patients as 1 patient had 2 primary tumors (Figure 1). The most common site of primary tumor was pancreas followed by duodenum (Table 2). In the remaining 2 patients, primary tumor was not localized by planar scintigraphy or SPECT/CT. The overall sensitivity, specificity, PPV, NPV, and accuracy of ^99m^Tc-Hynic TOC planar or SPECT/CT for primary tumor are detailed in Table 3. ^99m^Tc-Hynic TOC planar or SPECT/CT findings were true positive for primary tumor in 14 patients, It localized primary tumor in 8 patients with carcinoid, 4 patients with pancreatic NET, and 4 patients with NET not otherwise specified (NOS).

**Table 2:** Primary and metastatic sites obtained with Tc-99m HYNIC-TOC imaging

Finding		Conventional Imaging	Tc-99m HYNIC-TOC imaging
**Localised primary**		7	14
**Non localised primary**		9	2
**Sites of primary**	Stomach	0	1
	Duodenum	2	3
	Rectum or colon	1	1
	Pancreas	2	5
	Jejunum	1	2
	Ileum	1	1
	Others	0	1
	**Metastases**	18	22
	Liver	14	20
	Lymph node	4	6
	Bone	1	2
	Others	2	5

Most of the patients had more than one site of metastases.

**Table 3:** Patient-Based Diagnostic Accuracy of Conventional Imaging and scintigraphy for the Detection of Primary Tumor

Performance	Conventional Imaging %	Scintigraphy Imaging %
Sensitivity	43.75	87.5
Specificity	100	85.7
Positive Predictive value	100	93.3
Negative Predictive value	37.5	75
Accuracy	59.1	86.9

### ^99m^Tc-Hynic TOC planar or SPECT/CT for Metastases

**Figure 2:**
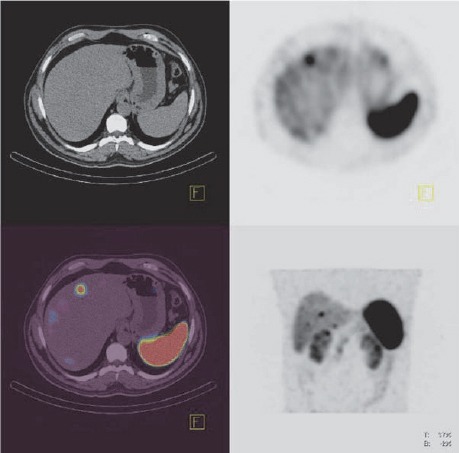
Transaxial SPECT, CT and fused SPECT/CT as well as coronal image shows uptake in the duodenum and liver lesions.

^99m^Tc-Hynic TOC planar or SPECT/CT detected metastases in all 22 patients who had one or more sites of metastases according to the reference standard. A total of 56 regions were noted in these 22 patients. The most common site of metastases on ^99m^Tc-Hynic TOC planar or SPECT/CT was the liver (Figure 2), followed by the lymph nodes (Table 2). ^99m^Tc-Hynic TOC planar or SPECT/CT findings were false negative for few of the liver metastases in 3 patient. The overall sensitivity, specificity, PPV, NPV, and accuracy of ^99m^Tc-Hynic TOC planar or SPECT/CT for detecting metastases are detailed in Table 4. ^99m^Tc-Hynic TOC planar or SPECT/CT was true-negative in 6 lesions and there was one false-positive lesion, when based on histopatholgy results or imaging follow up, where available.

**Table 4:** Patient-Based Diagnostic Accuracy of Conventional Imaging and SPECT/CT for the Detection of metastases

Performance	Conventional Imaging %	SPECT-CT Imaging %
Sensitivity	81.8	100
Specificity	60	85.7
Positive Predictive value	81.8	95.6
Negative Predictive value	60	100
Accuracy	75	96.5

### Semi quantitative Analysis

Semi quantitative analysis (tumor to background and tumor to normal liver uptake ratios) of primary tumors and metastases were evaluated in all lesions. Among the primary tumors, histologic subtype did not influence the uptake of ^99m^Tc-Hynic TOC planar or SPECT/CT. No significant difference was found in the uptake of carcinoid compared with pancreatic NET and NET NOS. Similarly, no significant difference was found between pancreatic NET and NET NOS. However, the tumor to background and tumor to normal liver uptake ratios of liver metastases was significantly higher than the uptake ratios of lymph node and bone metastases. No significant difference was found in uptake ratios between lymph node and bone metastases. Overall, no significant difference existed in uptake ratios of primary tumor and of metastases.

### Conventional Imaging for Primary Tumor

Conventional imaging detected primary tumor in 7 of 16 patients who had primary tumor according to the reference standard. The overall sensitivity, specificity, PPV, NPV, and accuracy of conventional imaging for primary tumor are shown in Table 3. Conventional imaging findings were true positive for primary tumor in 7 patients, true-negative in 6.

### Conventional Imaging for Metastases

**Figure 3:**
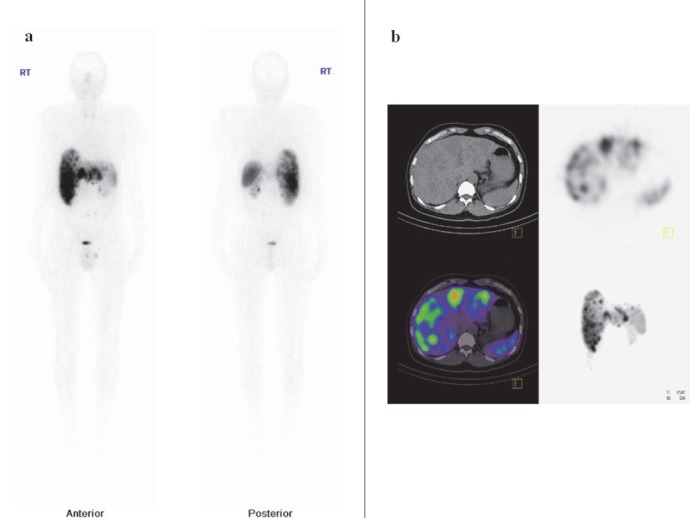
(a) Whole body anterior and posterior Tc-99m-HYNIC-TOC images showing multiple liver metastasis. (b) SPECT, CT and SPECT/CT localized uptake to the liver lesions.

Conventional imaging detected metastases in 18 of 22 patients who had one or more sites of metastasis according to the reference standard. A total of 34 lesions were noted in these 18 patients (Table 3). The most common site of metastases on conventional imaging was the liver (Figure 3), followed by the lymph nodes. The overall sensitivity, specificity, PPV, NPV, and accuracy of conventional imaging for detecting metastases are detailed in Table 4.

### Comparison of ^99m^Tc-Hynic TOC planar or SPECT/CT and Conventional Imaging for Primary Tumor

^99m^Tc-Hynic TOC planar or SPECT/CT detected primary lesions in 14 patients compared with 7 patients on conventional imaging. In 2 patients, additional primary sites were detected with ^99m^Tc-Hynic TOC planar or SPECT/CT. The total number of primary sites seen on ^99m^Tc-Hynic TOC planar or SPECT/CT was 15, whereas 7 primary sites were seen by conventional imaging.

### Comparison of ^99m^Tc-Hynic TOC planar or SPECT/CT and Conventional Imaging for Metastases

^99m^Tc-Hynic TOC planar or SPECT/CT detected metastases in 22 patients compared with 18 patients on conventional imaging. A total of 56 metastatic regions were seen on ^99m^Tc-Hynic TOC planar or SPECT/CT compared with 34 lesions on conventional imaging. In 8 patients, one or more additional metastatic sites were detected with ^99m^Tc-Hynic TOC planar or SPECT/CT. On analysis, ^99m^Tc-Hynic TOC planar or SPECT/CT was superior to conventional imaging for the detection of liver, lymph node and bone metastases.

### Impact of ^99m^Tc-Hynic TOC planar or SPECT/CT on Patient Management

Findings from this study led to a change in the management strategy in a number of patients. On the basis of the results, 6 patients (27.2%) underwent surgery for primary lesions that were not detected on other imaging modalities. In another 3 patients (13.6%), ^99m^Tc-Hynic TOC planar or SPECT/CT helped in surgical planning by depicting additional surgically resectable sites. 9 patients (41%) were spared unnecessary surgery because evidence of the advanced stage of disease was seen. It changed the treatment regimen in 8 patients with liver metastases and ruled out liver metastases in one patient. In addition, the detection of the expression of somatostatin receptors in these patients led to the continuation of octreotide-based treatments. In 4 patients with progressive disease, imaging findings of strong receptor positivity led to peptide-based radionuclide therapy with ^177^Lu DOTA-d-Phe(1)- Tyr(3)-Octreotate (TATE). Although continuation of treatment cannot be considered to be as management change, ^99m^Tc-Hynic TOC planar or SPECT/CT definitely helped in supporting the treatment decisions in these patients (70%).

## Discussion

In the past decade, SRS using ^111^In-pentetreotide (OctreoScan) has played a central role in the functional assessment of NETs with a high degree of sensitivity and acceptable specificity for both primary as well as metastatic gastroenteropancreatic NETs. However, problems with SPECT emanate from the low resolution of the system that hampers imaging lesions that are small or have low receptor densities (35, 36). More recently, the development of novel PET tracers (^68^Ga-DOTA peptides) that bind specifically to somatostatin receptors expressed on the surface of NET cells allows the visualization of NET on ^68^Ga-DOTA-peptide PET/CT scans. Several different DOTA peptides (DOTA-d-Phe(1)- Tyr(3)-Octreotide [TOC], DOTA-NOC, and DOTA-TATE) have been used in this clinical setting for either NET diagnosis or peptide receptor radionuclide therapy. However the use of Gallium based somatostatin scintigraphy is restricted to centers with a PET-CT facility and is additionally expensive to procure the generator and post elution technology. Hence, the search for a suitable gamma camera based alternative is mandatory, to enable universal utilization of somatostatin scintigraphy for diagnosis and staging as well as a prelude to therapy.

Decristoforo *et al* preclinical studies (32-34) show that ^99m^Tc-Hynic TOC has important features in common with Octreoscan, (i.e., fast plasma clearance and, predominantly, urinary excretion). Moreover, the new tracer has advantages deriving from the optimal physical characteristics of being labeled with ^99m^Tc, improved scintigraphic image quality, availability and lower cost. In addition, as demonstrated by Maecke and Béhé (27) and Decristoforo (32), ^99m^Tc-Hynic TOC is characterized by a higher uptake in tumors than that of Octreoscan, especially when compared to the muscles and the heart.

**Figure 4:**
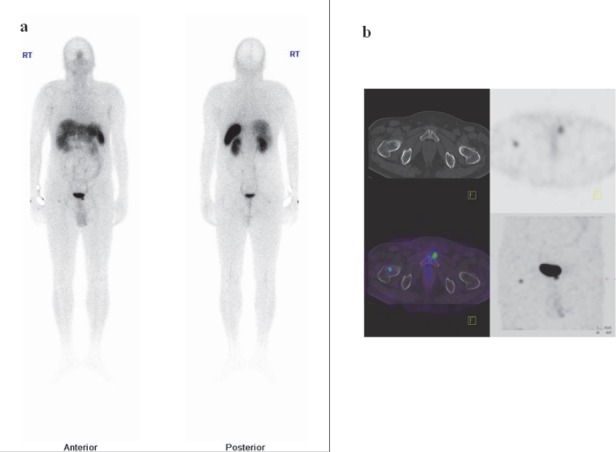
(a)Whole body Tc-99m-HYNIC-TOC images showing two focal uptake, one in the right thigh and other just below bladder. Also photopenic area noted in the liver lesion suggestive of necrosis.(b) Transaxial SPECT, CT and SPECT/CT showing uptake in the neck of right femur and left pubis.

Several studies in the past have established the efficacy of this tracer in NETs with varying results. In the current study, ^99m^Tc-Hynic TOC planar or SPECT/CT showed a sensitivity of 87.5% and specificity of 85.7% for primary tumor. Traditionally pancreatic NETs are usually well differentiated with a higher expression of somatostatin receptor subtype 2A (37, 38). Most patients in the present study had well differentiated tumors with low Ki67 indices and differentiation grade 1-2, which might explain the high uptake even in metastatic lesions. The sensitivity and specificity of ^99m^Tc-Hynic TOC planar or SPECT/CT for metastatic disease was 100% and 86%, respectively. This high sensitivity is consistent with high affinity somatostatin binding sites which have been found *in vitro* on most gastroenteropancreatic endocrine tumors (39, 40). The majority of these tumors contain high numbers of receptors homogeneously distributed throughout the tumor site. There was one false-positive study for primary tumor which was later confirmed to be physiologically high uptake in the uncinate process of the pancreas. Recent reviews routinely mention this finding of increased uptake in the head of pancreas as physiologic (41). ^99m^Tc-Hynic TOC planar or SPECT/CT showed higher accuracy for both primary (87% vs 60%) and metastatic gastroenteropancreatic NETs (97% vs 75%) in comparison to conventional imaging modalities (*P*<0.0001). In seven patients, additional primary sites were detected with ^99m^Tc-Hynic TOC planar or SPECT/CT. On lesion analysis, ^99m^Tc-Hynic TOC planar or SPECT/CT was superior to conventional imaging for the detection of lymph node (*P*<0.0001) and bone (*P*=0.002) metastases (Figure 4). However, no significant difference was found for liver metastases (*P*=1.000). In two patients with documented liver metastases ^99m^Tc-Hynic TOC planar or SPECT/CT showed no uptake. This lack of uptake could be the result of poor differentiation of the lesions. Negative ^99m^Tc-Hynic TOC planar or SPECT/CT findings are also crucial to select the appropriate therapy with combined chemotherapy using etoposide and cisplatin (42). In the current study, ^99m^Tc-Hynic TOC planar or SPECT/CT had a significant impact on the management of patients with gastroenteropancreatic NETs. There was a substantial change in the management protocol for 6 patients (32%). 2 patients underwent surgery for primary lesions that were not detected on other conventional imaging modalities. One patient had confirmation of a primary tumor in the pancreas, which was suspected on conventional imaging. Other patients underwent resection of primary tumor based on ^99m^Tc-Hynic TOC imaging that was not detected on other modalities.

In 3 patients, ^99m^Tc-Hynic TOC planar or SPECT/CT assisted in the evaluation of locoregional extension, which helped the surgeon to plan curative surgery. In 1 of these patients, additional primary foci were detected for surgical removal. In two patients, additional nodal disease was detected, which led to complete excision. 2 other patients were spared unnecessary surgery for the advanced stage of disease. In one patient with a primary pancreatic gastrinoma, surgery was delayed because of ^99m^Tc-Hynic TOC upstaging with detection of liver metastases. Thus, there was an overall influence in almost half of the patients (42%) regarding management decisions (change in 32% and support in 10%).

There have been reports of a kit formulation for the preparation of ^99m^Tc-Hynic TOC by Guggenberg *et al* (44). Though very simple to formulate, there were some limitations such as the lack of commercial kits in India, as well as a maximum of only two patient doses could be prepared using a single vial. The BARC kit is more versatile with capability of handling higher volumes of Technetium eluate, single step addition with no separate requirement for buffer solution, capability of handling up to 80 mCi (2,960 MBq) without compromising on radio chemical purity or image quality. The demonstration of quality of prepared product as well as high clinical performance in GPNET population will encourage ^99m^Tc-Hynic TOC application in neuro endocrine patients for staging and planning treatment. Especially in developing countries, where the patient load is high but centers with a PET CT facility or Germanium Gallium based imaging facilities are very few; this would be economically a much more viable alternative with equivalent diagnostic sensitivity and specificity. More investigations comparing the SPECT and PET radiopharmaceuticals are needed to further establish ^99m^Tc-Hynic TOC based imaging in the clinical algorithm.

A few limitations of the current study should be kept in mind. First, histopathologic confirmation of all lesions was not available. This was not technically or ethically feasible. Second, we could not compare the results with PET based somatostatin scintigraphy; which is currently considered the standard of care. Third, we did not attempt to reevaluate the conventional imaging modalities studies. Hence, we cannot comment on retrospective visibility of any lesion that was initially missed.

## Conclusion

The newly developed lyophilized kits from BARC performed well in the clinical setting. ^99m^Tc-Hynic TOC planar or SPECT/CT appears to be a highly sensitive and specific modality in the detection of gastroenteropancreatic NET. It is better than conventional imaging for this patient population and can have significant impact on patient management. The detection of a high degree of somatostatin receptor expression can be exploited to treat patients with cold octreotide therapy and peptide receptor radionuclide therapy, while a negative finding on ^99m^Tc-Hynic TOC planar or SPECT/CT can guide the physician to choose an alternate form of treatment.
